# Five-year trend analysis of malaria prevalence in Shewarobit, Amhara Regional State, North-central Ethiopia

**DOI:** 10.11604/pamj.2021.40.237.30614

**Published:** 2021-12-17

**Authors:** Tadegew Teshome Shiferawu, Azene Tesfaye Desta

**Affiliations:** 1 Department of Zoological Sciences, College Natural Sciences, Addis Ababa University, Addis Ababa, Ethiopia,; 2 Department of Medical Laboratory Science, College of Medicine and Health Sciences, Arba Minch University, Arba Minch, Ethiopia

**Keywords:** Trend, malaria, *plasmodium* species, shewarobit

## Abstract

**Introduction:**

analysis of the prevalence of malaria infection in health facilities is crucial for transmission dynamics and implementing evidence-based control strategies. The study was to determine a five-year pattern of malaria infection in Shewarobit, Northcentral Ethiopia.

**Methods:**

institutional based retrospective study was carried out to determine the prevalence of malaria infection from a five-year examination of malaria cases at Shewarobit Health Center, Ethiopia. The directory of all malaria cases reported between 2013-2017 was carefully examined and recorded. Data were analyzed using SPSS version 20.0 and the results were presented in tables and figures.

**Results:**

the results confirmed a total of 33,932 malaria suspects were diagnosed using microscopy over the last 5 years, of which 4705 (13.9%) were positive for malaria infection. Out of 4705 positive individuals, 3074 (65.3%) were males and 1631 (34.7%) were females. Plasmodium vivax, Plasmodium falciparum, and mixed infection (both species) accounted for 44.8%, 44.1%, and 7.1% of the confirmed cases, respectively.

**Conclusion:**

the study demonstrated that malaria infection is a public health concern in the study area, and Plasmodium vivax was the predominant species. Thus, the district health bureau and other concerned stakeholders should strengthen evidence-based intervention of malaria control strategies to eliminate malaria infection.

## Introduction

Malaria is a protozoan disease caused by parasites of the genus *Plasmodium* [1]. It is one of the leading causes of illness and death in the world [2]. According to the 2016 World Malaria Report, about 119,814 people were died in the world by malaria in 2015, among these 117,886 deaths occurred in Africa. About 453,000 malaria deaths were estimated to occur in children aged less than five years that were 78% of the global total deaths [3, 4]. It is one of the major health issues in Ethiopia [5]. The different agroecological contribute to malaria transmission, and it makes the distribution pattern seasonal and irregular, commonly characterized by predominant focal and widespread epidemics [6].

The burden of malaria continues to cause a substantial number of morbidity and mortality which accounts for most outpatient visits, and it has been one of the main causes of hospitalization and deaths in the country [7, 8]. Approximately 60% of Ethiopia´s population lives in malarious areas, and 68 % of the country´s landmasses are favorable for malaria transmission, with malaria primarily associated with altitude and rainfall [9]. The transmission of malaria in Ethiopia is seasonal and uneven. The transmission peaks bi-annually from September to December and from April to May, with higher transmission rates in the former period. The transmission is corresponding with the major harvesting periods in rural areas. This could lead to a severe economic burden for the country in different ways [7, 9]. *P. falciparum* and *P. vivax* are the dominant parasites responsible for the majority of malaria cases in Ethiopia [6, 10]. This proportion varies from place to place and from season to season. *P. falciparum* is the dominant parasite species in malaria epidemic situations, and this species causes severe and complicated manifestations and almost all malaria deaths [6].

Effective malaria control strategies were implemented in different parts of the country [11], there is scanty information on the trends of malaria infections, particularly in the study area. Furthermore, current information regarding gender, age, and locality-based prevalence of malaria are inadequate in the study area despite the identification of the disease burden since the early of the last century. To the best of our knowledge, investigation of the trends of malaria infection is necessary to design and implement effective malaria control strategies in the study area. To this end, the study aimed to determine the five-year pattern of malaria infection prevalence in Shewarobit, Northcentral, Ethiopia, from 2013-2017.

## Methods

### Study area

The study was conducted in Shewarobit town which is located in North Shewa Zone, Amhara regional state, Northcentral Ethiopia. It is located 225 km to the North of Addis Ababa with geographic coordination of 9°50'16" and 10°01' 32" North latitude and 39° 52' 58", and 39° 54' 9" East longitude with an area of 992.5 hectares in the rift valley with an altitude of 1280 meters above sea level. According to the City Administration Agriculture Office document, the geography of the town is mainly plain (79%), plateau (15.5%), up and down (3%), and swamp area (2.5%). The climate is tropical and has an annual temperature range from 28-37°C and the annual rainfall of the town is 1000 mm. The main rainy season usually occurs from June to September. Demographic data revealed that the town has nine kebeles with a total population of about 50,528 of which 24,638 (48.8%) were men and 25,890 (51.2) were women (Source, Town vital Event Registration Office 2018). Most of the dwellers of Shewarobit town are merchants, employers, daily laborers, and urban farmers. All Kebeles are malarious and infection is the most prevalent seasonal disease in the area in health center and September up to November and from June to August, October to December is the climax malaria infection transmission season in the area. Both *P. vivax* and *P. falciparum* exist in the area with widespread throughout the year.

### Study design and population

A health facility-based retrospective study was conducted to determine the trends of malaria burden during the past 5 years at Shewarobit Health Center. The target populations for the study were all malaria suspected individuals who had visited the health center from January 2013 to December 2017.

**Inclusion and exclusion criteria:** since it was retrospective study, a total of the five years (January 2013 to December 2017) suspected and confirmed individuals with malaria cases reports were included in the study, and all incomplete data and many febrile cases that possibly received treatment either from health extension workers or self-medication were excluded.

### Data collection

A five-year (2013-2017) retrospective data on the trend of malaria prevalence were carefully reviewed from the institutional malaria registration book. The parameters such as date of examination, total clinically treated and confirmed cases in months and years, types of malaria species, and socio-demographic data such as age and sex were collected. Data were collected by experienced medical laboratory technicians. Any data such as socio-demographic, and malaria diagnosis results that were not properly documented were excluded. Throughout the reviewed period, microscopy was used as the gold standard for the detection and species identification of Plasmodium parasites by examination of peripheral smears of stained blood films, as per the WHO protocol. The health center strictly follows the standard operating procedures for capillary and venous blood sample collection, smear preparation, staining, and blood film examination for malaria parasite detection in all phases of quality control. The blood slides were read and then classified qualitatively as either negative, *P. falciparum* positive, *P. vivax* positive, or mixed infection. Patients with positive peripheral blood smears were offered anti-malarial treatment as per national guidelines [12].

### Operational definition

**Malaria suspected individual:** any person with a fever or fever with headache, chills, rigor, back pain, sweats, myalgia, nausea, and vomiting arebeing diagnosed clinically as malaria [13].

**A confirmed malaria case:** is any suspected case of malaria confirmed by microscopy for the Plasmodium parasite [13].

**The population at risk:** population living in a geographical area in which locally acquired malaria cases occurred in the current and/ or previous years.

**Malarious area:** area which is located at an altitude level below 2,000 meters above sea level [14].

### Factors affecting the pattern of malaria infection

During malaria data collection, any malaria intervention activities that had been taken in each year to control malaria were collected using a well-prepared checklist. There was increased attention to malaria control and preventive activities by different responsible bodies, increased awareness of the community on the use of insecticide-treated bed nets (ITNs), and other malaria control activities through health education, increased accessibility of ITNs to the community, the increment of a budget for malaria control and prevention activities (personal communication with the head of the health center). But in the study area, there were no special factors that attributed to the increased or decreased occurrence of malaria cases.

### Data quality control

The completeness of the malaria registration books in the health center was first assessed to ensure the quality of data. Then, a data collection format sheet was prepared and used for data recording. Before data extraction, data collectors were adequately trained about the data extraction. The overall process of data extraction was followed up by the investigators, where a sample of completed data collection forms was randomly selected and checked daily for accuracy, completeness, and consistency. We have also checked the number of confirmed cases with the number of suspected cases throughout the reviewed data.

### Data processing and analysis

Data were entered using Epi Data 3.1 and analyzed using Statistical Package for the Social Science (SPSS 20.0). Descriptive statistics were employed to calculate frequencies and percentages of overall malaria cases, trends of malaria transmission in terms of years, *Plasmodium* species, gender, age, and distribution by season (months). The Pearson´s Chi-square test was used to describe the associations of variables. Test of significance was estimated assuming a at 0.05 and a p-value less than 0.05 was considered significant. Findings were summarized using tables, line graphs, and bar charts.

### Ethics consideration

Five-year retrospective data were collected from the health centers after ethical clearance was obtained from the College of Natural Sciences Institutional Ethics Review Board, Addis Ababa University, and submitted to Shewarobit Health Center, where all information gathered from the recorded data. After discussing the purpose and method of the study, a permission letter to use the data and conduct the study was obtained from the administrative office of the health center with the reference number S/R/H/C/44/017. Confidentiality of the information gathered from the surveillance database was assured, and all information was used for this study only.

## Results

### Annual trends of malaria infection prevalence

**Figure 1:**
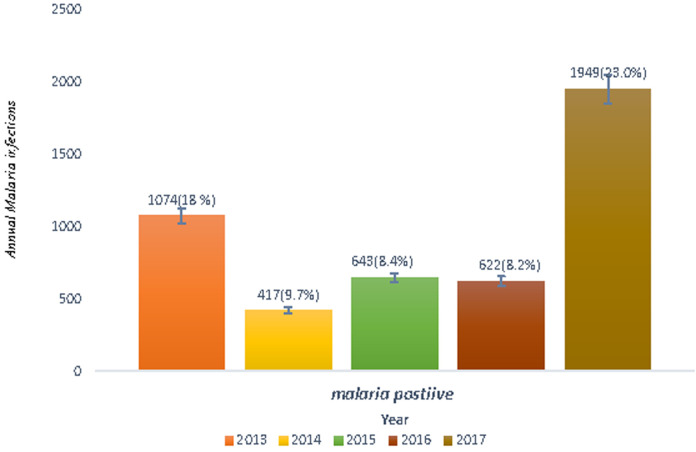
annual trends of malaria infection in Shewarobit Northcentral Ethiopia (2013-2017)

Within the last five years (2013-2017), a total of 33,932 blood films were requested for malaria diagnosis in Shewarobit Health Center. From these requested blood films, 4705 (13.9%) outpatients were microscopically confirmed as malaria positive. There were fluctuating trends of malaria within the last five years with the minimum 622 (8.2%) microscopically confirmed malaria positive were reported in 2016 and the maximum 1949 (23.0%) microscopically confirmed malaria positive were reported in 2017 in the study area. The data showed that malaria infection decreased from 18.0% in 2013 to 8.2% in 2016, but the infection increased in 2017 (23.0%) (Figure 1).

### Seasonal variation of malaria infection

**Figure 2:**
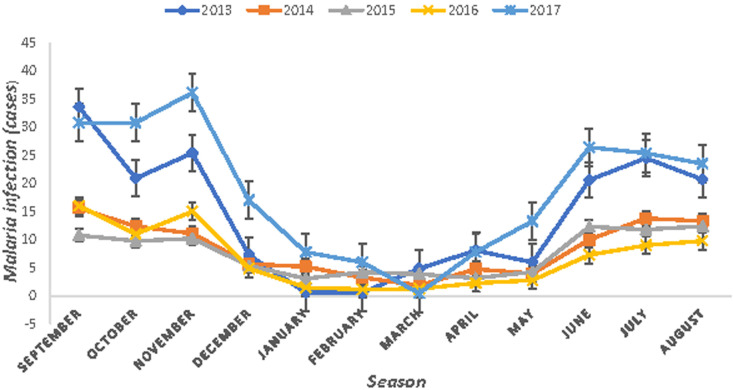
seasonal variation of malaria infection in Shewarobit, Northcentral, 2013-2017

Even though there were fluctuations in malaria infection and, malaria cases occurred in almost every month of the year in the study area. The highest peak of malaria infection was mainly recorded from September up to November months in all years, followed by the months of June up to August and the minimum numbers of malaria cases were recorded during the months of December up to May in the study area (Figure 2).

### Prevalence of malaria infections in relation to sex

**Figure 3:**
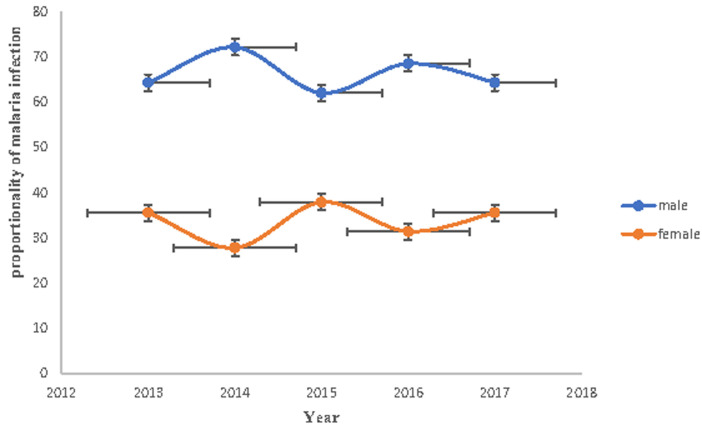
malaria infection prevalence in relation to sex in Shewarobit, Northcentral Ethiopia, 2013-2017

According to the record review, in the last five years (2013-2017) in the study area, males were more affected than females by malaria infections each year. Out of 4705 positive outpatients, 3074 were males and 1631 were females. The infection rates among males were 65.3% and females were 34.7%. The highest infections of males were occurred in 2014 (72.2%) and females were occurred in 2015 (37.9%) and the least infections of males were occurred in 2015 (62.1%) and females were in 2014 (27.8%) (Figure 3).

### Prevalence of malaria infections in relation to age groups

**Figure 4:**
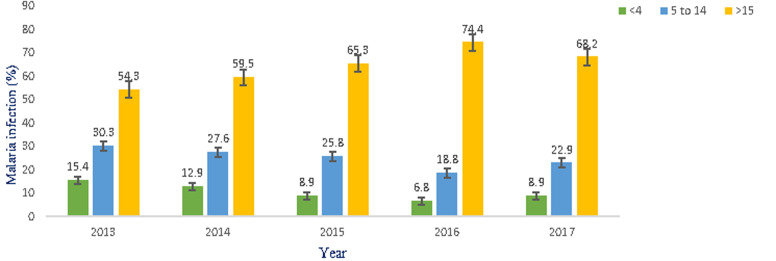
malaria infection prevalence in relation to age group in Shewarobit, Northcentral Ethiopia, 2013-2017

The age-specific prevalence rate of malaria was 492 (10.5%) for the age group ≤ 4 years old, 1170 (24.9%) for the age group between 5-14 years old, and 3043 (64.6% for the age group ≥ 15 years old. Although malaria infections were reported in all age groups, the most susceptible age groups were ≥ 15 years old with an overall prevalence of 64.6%, the next susceptible age groups were from 5 to 14 years old with a total prevalence of 24.9% and the least susceptible age groups were ≤ 4 years old with the total prevalence 10.5%. In the age group ≤ 4 years old, the highest infections were occurred in 2013 (15.4%) and the least infections were occurred in 2016 (6.8%). For the age groups between 5 and 14 years old, the highest infections were occurred in 2013 (30.3%), and the least infections occurred in 2016 that (18.8%), and for the age group =15 the highest infections were occurred in 2016 (74.4%) and the least infections were occurred in 2013 (54.3%) in the study area (Figure 4).

### Distribution of *Plasmodium* species

**Figure 5:**
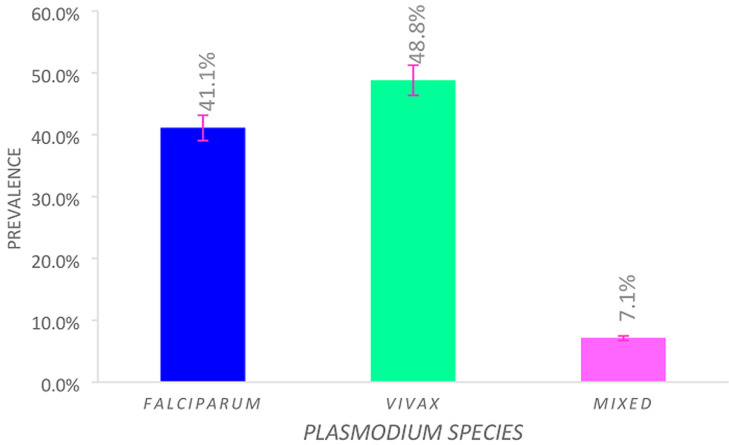
distribution of *Plasmodium* species in Shewarobit, Northcentral Ethiopia (2013- 2017)

The patterns of malaria infections by different species of Plasmodium parasites in the study area were indicated. 2296 (48.8%) of the outpatients were infected by *P. vivax*, followed by 2077 (41.1%) by *P. falciparum* and 332 (7.1%) were infected by mixed infection. The dominant *Plasmodium* species in the study area was *P. vivax* (Figure 5).

### Patterns of Plasmodium infection in relation to sex and age groups

The data showed that in all years males were more affected by *P. falciparum, P. vivax*, and mixed infections than females. The outpatients were highly infected by *P. falciparum*in 2015 (63.6%), among these 272 (66.5%) were males and 137 (33.5%) were females. The outpatients were highly infected by *P. vivax* in 2013 (78.5%), among these 548 (65%) were males and 295 (36%) were females, and the outpatients were highly infected by a mixed infection in 2017 (10.1%) of these 141 (71.9%) were males and 55 (28.1) were females. Concerning the age groups, the age groups ≤ 4 years old were highly infected by *P. falciparum* in 2014 (12.1%), by P. *vivax* in 2013 (17.3%), and by mixed infection in 2015 (16.2%). The age groups between 5 to 14 years old were highly infected by *P. falciparum* in 2014 (27.6%), by *P. vivax* in 2013 (32.2%), and by mixed infection in 2016 (27.2%). Finally, the age groups ≤ 15 years old were highly infected by *P. falciparum, P. vivax*, and mixed infection in 2016 with the infection of 269 (76%), 178 (72.3%), and 16 (72.8%) respectively (Table 1).

**Table 1:** pattern of *Plasmodium* infection in relation to sex and age groups in Shewarobit, 2013-2017

Year and p. species	Total confirmed & (%)	Age								
		≤ 4 years old			5 to 14 years old			≥ 15 years old		
		M (%)	F (%)	T (%)	M (%)	F (%)	T (%)	M (%)	F (%)	T
2013 (n= 1074)										
P. falciparum	179(16.7)	10(5.6)	5(2.8)	15(8.4)	21(11.8)	18(10.0)	39(21.9)	78(43.6)	47(26.3)	125(69.8)
P. vivax	843(78.5)	86(10.2)	60(7.1)	146(17.3)	169(20.0)	103(12.2)	272(32.2)	293(34.8)	132(15.7)	425(50.4)
Mixed	52(4.8)	2(3.8)	2(3.8)	4(7.6)	9(17.3)	5 (9.6)	14(26.9)	24(46.1)	10(19.2)	34(65.3)
2014 (n=417)										
P. falciparum	156(37.4)	11(7.0)	8(5.1)	19(12.1)	25(16.0)	18(11.5)	43(27.6)	64(41.0)	30(19.2)	94(60.2)
P. vivax	230(55.2)	26(11.3)	7(3.0)	33(14.3)	51(22.1)	14(6.1)	65(28.2)	98(42.6)	34(14.8)	132(57.3)
Mixed	31(7.4)	2(6.5)	0(0)	2(6.5)	6(19.4)	1(3.2)	7(22.6)	18(58.1)	4(12.9)	22(71.0)
2015 (n=643)										
P. falciparum	409(63.6)	17(4.2)	6(1.5)	23(5.7)	68(16.6)	40(9.8)	108(26.4)	187(45.7)	91(22.2)	278(67.9)
P. vivax	203(31.6)	13(6.4)	16(7.9)	29(14.3)	18(8.9)	32(15.8)	50(24.7)	76(37.4)	48(23.6)	124(61.0)
Mixed	31(4.8)	3(9.7)	2(6.5)	5(16.2)	5(16.1)	3(9.7)	8(25.8)	12(38.7)	6(19.4)	18(58.1)
2016 (n=622)										
P. falciparum	354(56.9)	15(4.2)	4(1.1)	19(5.3)	40(11.3)	26(7.3)	66(18.6)	187(52.8)	82(23.2)	269(76)
P. vivax	246(39.5)	16(6.5)	7(2.8)	23(9.3)	27(11.0)	18(7.3)	45(18.3)	127(51.6)	51(20.7)	178(72.3)
Mixed	22(3.6)	0(0)	0(0)	0(0)	5(22.7)	1(4.5)	6(27.2)	10(45.5)	6(27.3)	16(72.8)
2017 (n=1949)										
P. falciparum	979(50.2)	42(4.3)	51(5.2)	93(9.5)	108(11,0)	88(9.0)	196(20.0)	450(46)	240(24.5)	690(70.5)
P. vivax	774(39.7)	28(3.6)	30(3.9)	58(7.5)	117(15.1)	69(8.9)	186(24.0)	369(47.7)	161(20.8)	530(68.5)
Mixed	196(10.1)	13(6.6)	10(5.1)	23(11.7)	50(25.5)	15(7.7)	65(33.2)	78(39.8)	30(15.3)	108(55.1)

* n = number of malaria confirmed cases, M = male, F = female, T = total

## Discussion

The results of the study revealed that the prevalence of malaria infection fluctuated each year. A total of 33,932 blood film samples were collected and tested for malaria diagnosis in Shewarobit Health Center (2013 - 2017) of which 4705 (13.9%) of the samples were microscopically confirmed as malaria positives. The data confirmed that malaria infection was decreased from 2013 (18.2%) to 2016 (8.2%). However, the highest malaria cases were recorded in 2017 (23.0%). This might be due to the proper implementation of malaria control strategies. However, high malaria infection was recorded in 2017 (23.0%) might be due to the creation of mosquito breeding sites (stagnant water) as a result of unfinished construction. The prevalence of malaria infection in Shewarobit Health Center was 13.9%. It is higher than the results of other studies conducted in Woreta Health Center 5.4% [15], Arsi Negelle Health Center 11.45% [16], but it is less than the results of other studies done in Kola Diba Health Center 39.6% [7], Hallaba Health Center 82.84% [17] and Wolaita Zone 33.27% [18]. This difference may be due to the type of study design used, climatic condition, malaria diagnosis technique, and the skill of the laboratory technicians. Our most intriguing finding is that males were more infected than females by malaria infection each year. Out of 4705 positive outpatients, 3074 were males and 1631 were females. The infection rate among males was 65.3% and females were 34.7%. These results agree with existing studies in Aresi Negelle Health Center [16], Kombolcha [19], Kola Diba [7] reported the prevalence of malaria infection among males was 55.4%, 44.6%; 68.1%, 31.89% and 52.6%, 47.3% respectively. This might be due to the lifestyle and occupation of males. Males are usually involved in agricultural activities during the infection season of malaria, they do not use long-lasting insecticidal nets properly as compare to their counterparts.

The highest peak of malaria infections was recorded in September, October, and November months of the year followed by June, July, and August, and the minimum numbers of malaria cases were recorded from December to May in the study area. This might be due to the environmental and climatological situations that permit the continual breeding of vectors in permanent breeding sites during these months. The result of this study is in line with the study findings in Aresi Negelle Health Center [16], Woreta Health Center [15], Kombolcha [19], Kola Diba [7], Butagira [20], Wolaita Zone [21]. One of the most significant findings in the research was malaria infection was 65.5%, 25.3%, and 10.6% in the aged groups of > 15 years, 5 - 14 years old ≤ 4 years old respectively. The results of the study were also in agreement with the study findings reported in Woreta Health Center [5], patients in the age group of 15 years old were 1.9 times more likely to be positive for malaria than individuals under the age of 5 years old, in Kombolcha [19], the highest malaria prevalence ( 69.69%) was seen in the 15 - 45 years age group, in Kola Diba [7], malaria infection was reported in a higher rate in the age group of 15-44 years old with a prevalence rate of (50.1%). Although malaria infection was reported in all age groups, the most vulnerable age groups were ≥ 15 years old with an overall prevalence of 65.5%. This might be related to their frequent outdoor activities like agriculture associated with irrigation, staying with agricultural practices, especially during the high transmission period. However, these findings are in contrast with the findings of previous research conducted in different parts of Ethiopia. These are Aresi Negelle Health Center [18], reported children in the age range 0 to 5 years old were the most affected groups by the disease, in Jimma town [22] higher malaria prevalence rate was observed among under-five children (11%), children in the age groups between 10 and 14 years were the most affected by the disease, in Ethiopia, positive malaria diagnosis rate decreased with age in Hallaba Health Center [17], the highest prevalence of malaria was seen in the age groups of 0-19 years old.

One of the most important findings relates to the distribution of *Plasmodium* species with the predominant species were *P. vivax*, which accounted for 2296 (48.8%) of the overall prevalence followed by *P. falciparum*, accounting for 2077 (44.1%) and mixed infection accounted for 332(7.1%). The results of the study coincide with the study findings reported from Aresi Negelle Health Center [16] *P. vivax* (74%), P. *falciparum* (19.8%) and (6.2%) mixed infection, in Hallaba Health Center [17], *P. vivax* (70.41%), P. *falciparum* (23.08%) and (6.51%) mixed infection, in butajira [20], Butajira area [23] *P. vivax* (86.5%), P. falciparum (12.4%) and (1.1%) mixed infection. The reasons why *P. vivax* dominates *P. falciparum* might be due to the relapsing characteristics of *P. vivax* at the time of relatively malaria-free seasons, climate variability, and developed resistance to the currently used drug chloroquine [7]. The results of the study contradict the results of other studies conducted in Woreta health center [22], which reported the prevalence of *P. falciparum* 53.7%, *P. vivax* 42.4%, and 3.9% mixed infection, in Kombolcha which reported the prevalence of *P. falciparum* 60.2%, *P. vivax* 35.5% [19], in Jiga area in North West Ethiopia [24] which reported nearly similar existence of *P. falciparum* and *P. vivax*, in South West Ethiopia which reported the prevalence of *P. falciparum* 54.6%, *P. vivax* 41.6% and 3.8% mixed infection (both species) [25]. The use of data from institutional-based retrospective studies provides an opportunity to monitor trends in malaria burden, socioeconomic inequalities, and potential equity gaps or gains as malaria control interventions are intensified over time. However, the data were collected from health facilities only and this might underestimate the actual burden of malaria in the community in the study area. Besides, this study was only limited to the quantitative aspects. Moreover, the trends of malaria mortality were not reported in this study.

## Conclusion

Malaria infection is a serious health problem in Shewarobit with the predominant of *P. vivax* followed by *P. falciparum*. Notably, there was a slight reduction of malaria infection from 2013 to 2016. However, malaria infection was high in 2017, and males of productive age were mostly affected which results in an economical loss. Thus, these findings call for urgent evidence-based interventions to eliminate the infection.

### What is known about this topic

- *Malaria is a serious health problem in most lowland areas of Ethiopia with the predominant species is Plasmodium falciparum followed by Plasmodium vivax;*
- *The distribution of malaria infection is seasonal and varies agroecological;*
- *The factors associated with malaria infection were varied from place to place and study setting*.

### What this study adds

- *The present study revealed that Plasmodium vivax is the predominant malaria infection in Shewarobit, Northcentral Ethiopia;*
- *The data showed that there was great variation in the distribution of malaria infection within each kebele of the Shewarobit town; The research also highlighted the current situation of the village has an impact on the distribution of malaria infection;*
- *The study also emphasized the age of the participants, living status, income level, long-lasting insecticidal nets, Indoor* Residual Spray, and mosquito breeding sites are the major determinants of malaria infection.

## References

[1] Dabaro D, Birhanu Z, Yewhalaw D (2020). Analysis of trends of malaria from 2010 to 2017 in Boricha District, Southern Ethiopia. Malaria Journal.

[2] Aschale Y, Mengist A, Bitew A, Kassie B, Talie A (2018). Prevalence of malaria and associated risk factors among asymptomatic migrant laborers in West Armachiho District, Northwest Ethiopia. Research and Reports in Tropical Medicine.

[3] Barber BE, Rajahram GS, Grigg MJ, William T, Anstey NM (2017). World Malaria Report: time to acknowledge Plasmodium knowlesi malaria. Malaria journal.

[4] World Health O (1991). Basic laboratory methods in medical parasitology: World Health Organization.

[5] Geleta G, Ketema T (2016). Severe malaria associated with Plasmodium falciparum and P vivax among children in Pawe Hospital Northwest Ethiopia. Malaria research and treatment.

[6] Alelign A, Tekeste Z, Petros B (2018). Prevalence of malaria in Woreta town, Amhara region, Northwest Ethiopia over eight years. BMC Public Health.

[7] Alemu A, Muluye D, Mihret M, Adugna M, Gebeyaw M (2012). Ten year trend analysis of malaria prevalence in Kola Diba, North Gondar, Northwest Ethiopia. Parasites & Vectors.

[8] Abate A, Degarege A, Erko B (2013). Community knowledge, attitude and practice about malaria in a low endemic setting of Shewa Robit Town, northeastern Ethiopia. BMC Public Health.

[9] FMOH (2015). Ethiopian National Malaria Indicator survey. Federal Ministry of Health.

[10] Feleke DG, Gebretsadik D, Gebreweld A (2018). Analysis of the trend of malaria prevalence in Ataye, North Shoa, Ethiopia between 2013 and 2017. Malaria journal.

[11] Addisu A, Tegegne Y, Mihiret Y, Setegn A, Zeleke AJ (2020). A 7-Year Trend of Malaria at Primary Health Facilities in Northwest Ethiopia. Journal of Parasitology Research.

[12] Ababa A (2004). Diagnosis and Treatment Guidelines for Health Workers in Ethiopia.

[13] Kalil FS, Bedaso MH, Wario SK (2020). Trends of Malaria Morbidity and Mortality from 2010 to 2017 in Bale Zone, Ethiopia: Analysis of Surveillance Data. Infection and Drug Resistance.

[14] Assefa A (2016). The third Ethiopian Malaria Indicator Survey 2015 (EMIS-2015) EPHA Conference Systems, 28th Annual conference.

[15] Derbie A, Alemu M (2017). Five years malaria trend analysis in Woreta Health Center, Northwest Ethiopia. Ethiopian Journal of Health Sciences.

[16] Hailemariam M, Gebre S (2015). Trend analysis of malaria prevalence in Arsi Negelle health center, Southern Ethiopia. Journal of Infectious Diseases and Immunity.

[17] Tefera G (2014). Prevalence of malaria and associated factors among patients attending at Hallaba Health Center, Southern Ethiopia. Immunol Infect Dis.

[18] Legesse D, Haji Y, Abreha S (2015). Trend analysis of malaria occurrence in Wolaita Zone, Southern Ethiopia: retrospective cross-sectional study. Malaria research and treatment.

[19] Gebretsadik D, Feleke DG, Fiseha M (2018). Eight-year trend analysis of malaria prevalence in Kombolcha, South Wollo, north-central Ethiopia: a retrospective study. Parasites & vectors.

[20] Tesfaye S, Belyhun Y, Teklu T, Medhin G, Mengesha T, Petros B (2012). Malaria pattern observed in the highland fringe of Butajira, Southern Ethiopia: a ten-year retrospective analysis from parasitological and metrological data. MWJ.

[21] WHO (2017). World Malaria Report.

[22] Alemu A, Tsegaye W, Golassa L, Abebe G (2011). Urban malaria and associated risk factors in Jimma town, south-west Ethiopia. Malaria journal.

[23] Woyessa A, Deressa W, Ali A, Lindtjørn B (2012). Prevalence of malaria infection in Butajira area, south-central Ethiopia. Malaria journal.

[24] Ayalew S, Mamo H, Animut A, Erko B (2016). Assessment of current malaria status in light of the ongoing control interventions, socio-demographic and environmental variables in Jiga Area, Northwest Ethiopia. PLoS One.

[25] Sena LD, Deressa WA, Ali AA (2014). Analysis of trend of malaria prevalence in south-west Ethiopia: a retrospective comparative study. Malaria journal.

